# Folate/NIR 797-Conjugated Albumin Magnetic Nanospheres: Synthesis, Characterisation, and *In Vitro* and *In Vivo* Targeting Evaluation

**DOI:** 10.1371/journal.pone.0106483

**Published:** 2014-09-04

**Authors:** Qiusha Tang, Yanli An, Dongfang Liu, Peidang Liu, Dongsheng Zhang

**Affiliations:** 1 Medical School, Southeast University, Nanjing, China; 2 Jiangsu Key Laboratory of Molecular and Functional Imaging, Department of Radiology, Zhongda Hospital, Medical School, Southeast University, Nanjing, China; King Abdullah University of Science and Technology, Saudi Arabia

## Abstract

A practical and effective strategy for synthesis of Folate-NIR 797-conjugated Magnetic Albumin Nanospheres (FA-NIR 797-MAN) was developed. For this strategy, Magnetic Albumin Nanospheres (MAN), composed of superparamagnetic iron oxide nanoparticles (SPIONs) and bovine serum albumin (BSA), were covalently conjugated with folic acid (FA) ligands to enhance the targeting capability of the particles to folate receptor (FR) over-expressing tumours. Subsequently, a near-infrared (NIR) fluorescent dye NIR 797 was conjugated with FA-conjugated MAN for *in vivo* fluorescence imaging. The FA-NIR 797-MAN exhibited low toxicity to a human nasopharyngeal epidermal carcinoma cell line (KB cells). Additionally, *in vitro* and *in vivo* evaluation of the dynamic behaviour and targeting ability of FA-NIR 797-MAN to KB tumours validated the highly selective affinity of FA-NIR 797-MAN for FR-positive tumours. In summary, the FA-NIR 797-MAN prepared here exhibited great potential for tumour imaging, since the near-infrared fluorescence contrast agents target cells via FR-mediated endocytosis. The high fluorescence intensity together with the targeting effect makes FA-NIR 797-MAN a promising candidate for imaging, monitoring, and early diagnosis of cancer at the molecular and cellular levels.

## Introduction

After the 2005 milestone of the clinical application of paclitaxel-albumin nanoparticles (NPs) (Abraxane) for the treatment of metastatic breast cancer [1], [2], albumin NPs are now well established as effective delivery systems in nanomedicine [3]. Albumin demonstrates several appealing characteristics, including biocompatibility, nontoxicity, *in vivo* metabolism into innocuous degradation products, non-immunogenicity, purification feasibility, water solubility, a long circulatory half-life, and a tendency to accumulate in tumours. Consequently, several laboratories are developing various albumin-based systems for the delivery of a variety of therapeutic and diagnostic compounds to tumours, including chemotherapeutic drugs, nanomedicines, cytokines, nucleic acids, radionuclides, fluorescent molecules, and many others [1], [4], [5].

Superparamagnetic iron oxide nanoparticles (SPIONs) have been attracting considerable interest as effective drug delivery systems, due to their simple and scalable preparation, specific magnetic properties, and biocompatibility [6]. SPIONs exhibit superparamagnetic behaviour at room temperature; i.e. they magnetise strongly under an applied magnetic field but fail to retain this property after the field is removed [7], [8]. This property makes them suitable for biomedical applications, such as targeted drug delivery [9], MRI contrast enhancement [10], [11], cancer diagnosis [12], hyperthermic treatment of tumours [13], [14], and magnetically mediated separation of biomolecules. Magnetohyperthermia is a particularly interesting application for SPIONs.

The development of tumour-specific imaging agents is highly desirable, as they can provide more accurate and earlier diagnoses, while also improving assessment of the biological aggressiveness of the tumour and monitoring of the treatment response [15]. The folate receptor (FR) displays specific characteristics that make it a promising target for tumour-specific imaging and therapy. FR, known as the high-affinity membrane folate-binding protein, is over-expressed in various human carcinomas, including ovarian, breast, colorectal, and nasopharyngeal, whereas its expression is low in normal tissue [16]. As a result, several folate conjugates have been prepared for targeted drug delivery [17], [18], [19], [20], [21] and folate-mediated diagnosis [22], [23]. Recently, *in vivo* early tumour diagnosis by near infrared (NIR) organic dye-folate conjugates [24] or folate-nanoparticle (Au magnetic nanoparticles) conjugates [25] was reported, which has opened up new possibilities for *in vivo* fluorescence imaging of tumours.

NIR fluorescence imaging at wavelengths of 700–900 nm has many advantages, including real-time *in vivo* monitoring of biological functions in living subjects, non-invasive whole-body imaging in small animals, and low absorption of light by intrinsic chromophores, such as haemoglobin and water, allowing light to penetrate deeper into tissues. Consequently, many NIR fluorescent contrast agents have been developed [24]. NIR fluorescent probes possess relatively low tissue absorption, scatter, and minimal autofluorescence [26], which results in greater tissue penetration than visible optical probes for *in vivo* imaging applications within the 650–900 nm NIR window. The NIR 797 isothiocyanate dye has absorption and emission maxima around 795 and 814 nm, demonstrating low absorptivity by tissue chromophores.

In the current study, we developed novel tumour-targeting nanospheres (FA-NIR 797-MAN), composed of NIR 797 isothiocyanate-folate-SPIONs-albumin nanospheres. Immunocytochemical studies of FA-NIR 797-MAN-binding activity in cultured cancer cells were performed to confirm the affinity between FRs and FA-NIR 797-MAN conjugates. These targeting nanospheres were injected into a subcutaneous KB xenograft mouse model. *In vivo* dynamic distribution and tumour targeting by FA-NIR 797-MAN were monitored using a near-infrared fluorescence imaging system.

## Results and Discussion

### Synthesis and characterisation of FA-NIR 797-MAN

**Figure 1:**
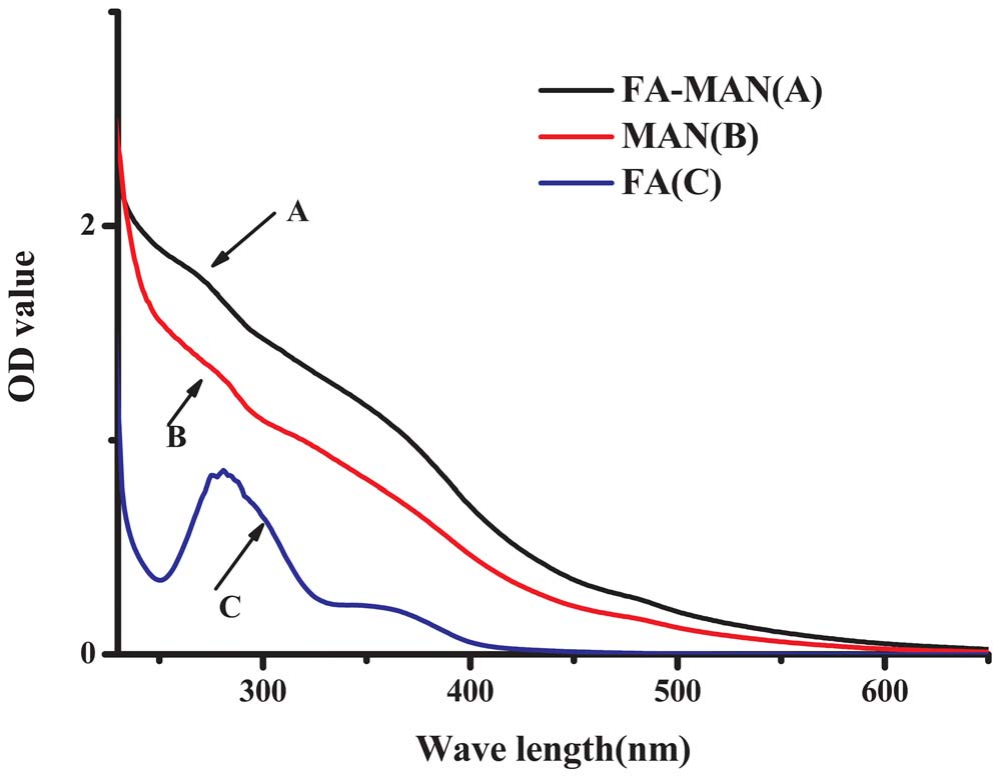
Absorption spectra of (A) FA-MAN, (B) MAN and (C) FA.

**Figure 2:**
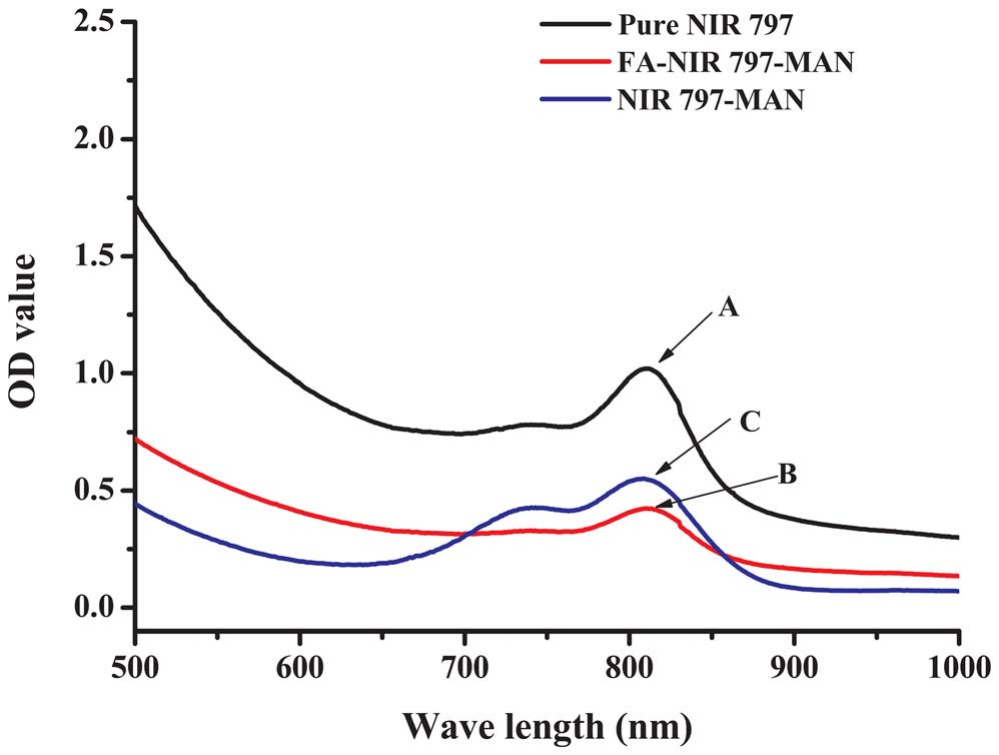
Absorption spectra of (A) NIR 797, (B) FA-NIR 797-MAN and (C) NIR 797-MAN.

**Figure 3:**
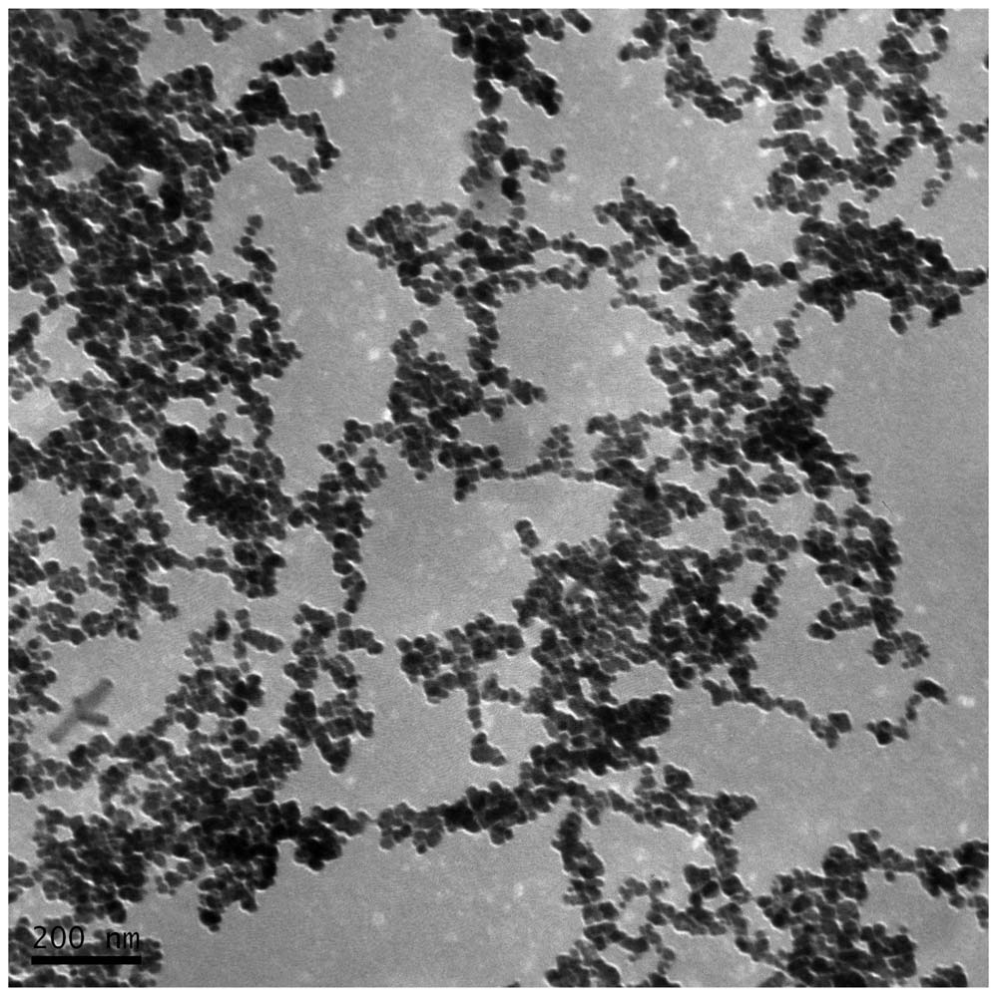
TEM image of FA-NIR 797-MAN nanospheres.

**Figure 4:**
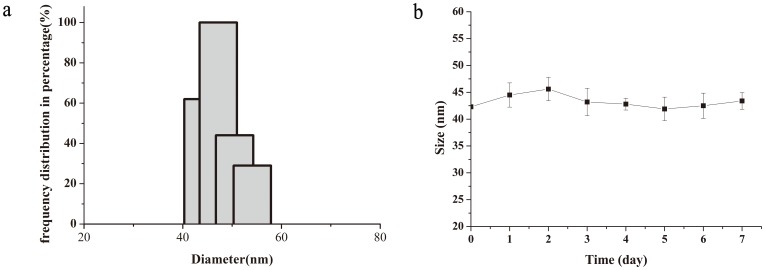
DLS measurement of FA-NIR 797-MAN nanospheres. (a) The graph shows size distribution of of FA-NIR 797-MAN nanospheres in water. (b) Average hydrodynamic diameter change of FA-NIR 797-MAN when incubating in water at 4°C for 7 days.

In this study, MAN was covalently conjugated with FA ligands to enhance the targeting capability of the particles to FR over-expressing tumours. The FA-NIR 797-MAN was prepared as described in the Experimental section. First, MAN was synthesised and conjugated with NHS-folate via an amide linker to yield FA-MAN. Second, NIR 797 was incorporated into the FA-MAN via an amide linker to yield FA-NIR 797-MAN. The UV spectra of FA-MAN, MAN, and FA are presented in Figure 1 A–C. Obviously; the folate peaks represent the predominant difference between FA-MAN and MAN. As shown in Figure 1, the characteristic peaks at 280 nm correspond to the folate present in FA-MAN; while MAN have no characteristic peaks in the 280 nm range. This confirmed the conjugation of folate with MAN in FA-MAN. The UV spectra of FA-NIR 797-MAN, NIR 797-MAN, and NIR 797 are presented in Figure 2 A–C. The UV spectrum of free NIR 797 was determined at 814 nm, and the characteristic peaks at 814 nm were seen in FA-NIR 797-MAN and NIR 797-MAN, indicating that NIR 797 conjugated with FA-NIR 797-MAN and NIR 797-MAN. Transmission electron microscopy (TEM) showed that FA-NIR 797-MAN are monodisperse, highly soluble, and stable in aqueous solution (Figure 3). The dynamic size of FA-NIR 797-MAN, as measured by DLS, was 48±4 nm (Figure 4a). As shown in Figure 4b, the average hydrodynamic diameter of FA-NIR 797-MAN was hardly changed when incubating in water at 4°C for 7 days.

**Figure 5:**
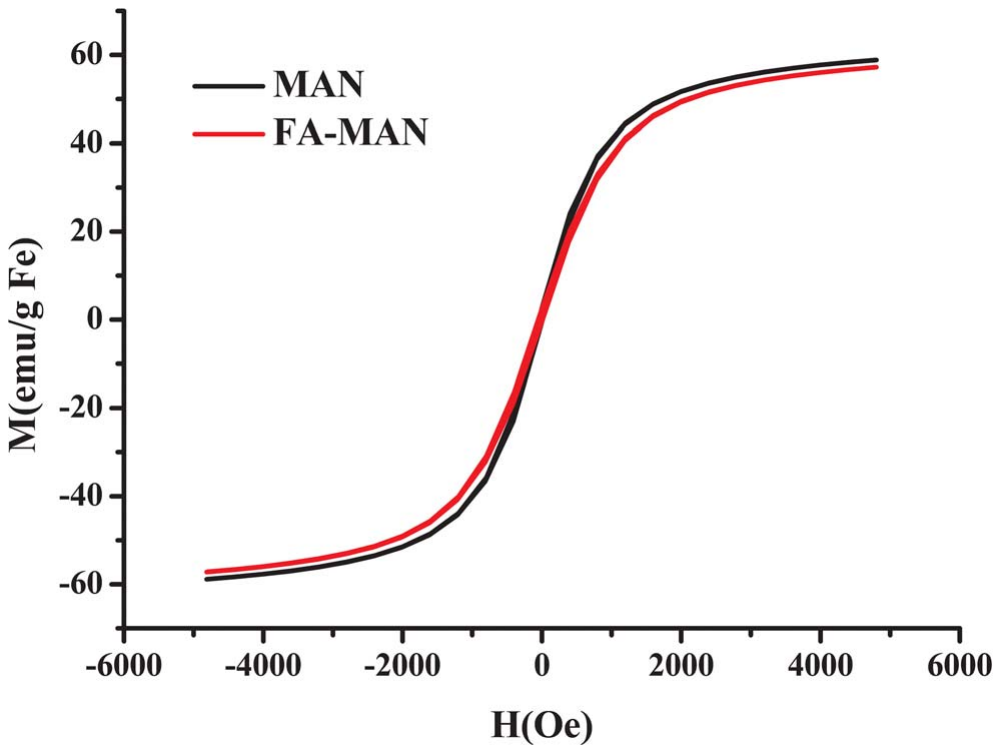
Magnetization curve as a function of field for MAN and FA-MAN at a temperature of 25°C.

For clinical application as a hyperthermic agent, it is critical that SPIONs retain their magnetic properties after the modification treatments. The magnetic properties of FA-MAN were investigated using the VSM with the MAN as a control. As expected, these SPIONs are superparamagnetic at room temperature, and the hysteresis loops showed negligible hysteresis. The saturation magnetisation values of MAN and FA-MAN at 25°C were 59 and 57 emu/g Fe, respectively (Figure 5), suggesting that the folate had little effect on the saturation magnetisation of the SPIONs.

### Cell toxicity assays

**Figure 6:**
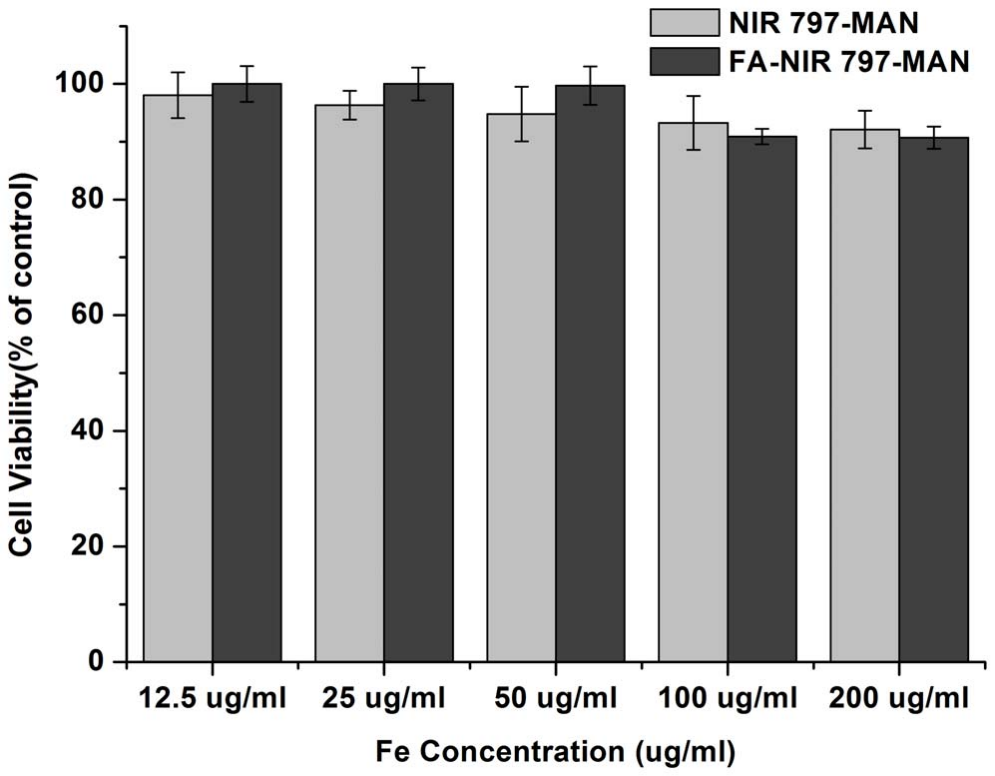
In vitro cytotoxicity of NIR 797-MAN and FA-NIR 797-MAN. Each data point shown is the mean ± SD from three independent experiments.

An MTT assay using KB cells was performed to evaluate the cytotoxicity of FA-NIR 797-MAN and NIR 797-MAN (Figure 6). KB cells were incubated for 24 h with FA-NIR 797-MAN and NIR 797-MAN at six concentrations from 12.5 to 200 µg Fe/mL. The cell viability measured by MTT assay was expressed as the fraction of viable cells in the cell population and normalised to that of cells that were not co-incubated with FA-NIR 797-MAN and NIR 797-MAN (blank control). As shown in Figure 6, 90% cell viability was retained after incubation as compared with the control. Therefore, the FA-NIR 797-MAN and NIR 797-MAN caused little to no cytotoxicity to the KB cells, even at the highest concentration used (200 µg Fe/mL). According to this result, these nanospheres represent potential non-cytotoxic probes for imaging applications.

### Nanosphere uptake by KB cells

**Figure 7:**
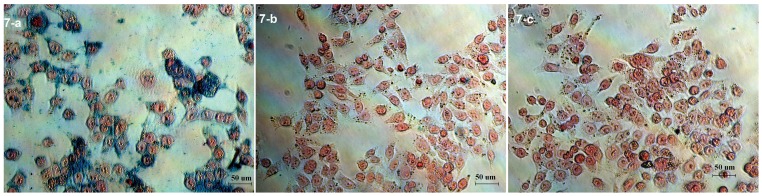
Prussian blue-stained KB cells after treatment with (a) FA-targeted group, (b) non-FA-targeted group and (c) FA-inhibited group (×400 magnification).

Since FA-NIR 797-MAN was developed to specifically bind to FR and trigger receptor-mediated internalisation in FR-positive cells [31], we compared the uptake by KB cells of each group of nanospheres: the FA-targeted, non-FA-targeted, and FA-inhibited. After a 24-h incubation with the nanospheres, followed by washing with PBS, the KB cells were stained with Prussian blue to quantitate intracellular iron oxide nanoparticles (Figure 7). Most of the KB cells that were incubated with the FA-targeted group (Figure 7-a) stained blue, indicating that the FA-targeted group was readily phagocytosed by the KB cells and remained predominantly in the cytoplasm. In striking contrast, little blue colour was detected in the cells incubated with the non-FA-targeted group(Figure 7-b) or the FA-inhibited group(Figure 7-c). The enhanced uptake of the FA-targeted group in FR-positive cancer cells suggests that this application could facilitate the diagnosis of FR-expressing cancers. The primary hypothesis of this study is strengthened by the tumour targeting capability of MAN, which was permitted by modification of the surface bioactivity of the nanospheres by FA. Retnakumari et al. [32] reported that without FA conjugation the BSA–Au did not show any specific attachment to MCF-7, whereas FA-conjugated Au–BSA NCs displayed enhanced uptake that resulted in red fluorescent staining of the cell membrane. This result is consistent with the high FR expression that we observed in KB cells. In addition, we compared in this study the cellular uptake of the FA-targeted, the non-FA-targeted, and the FA-inhibited groups, a subject on which few reports are available.

### *In vivo* optical imaging

**Figure 8:**
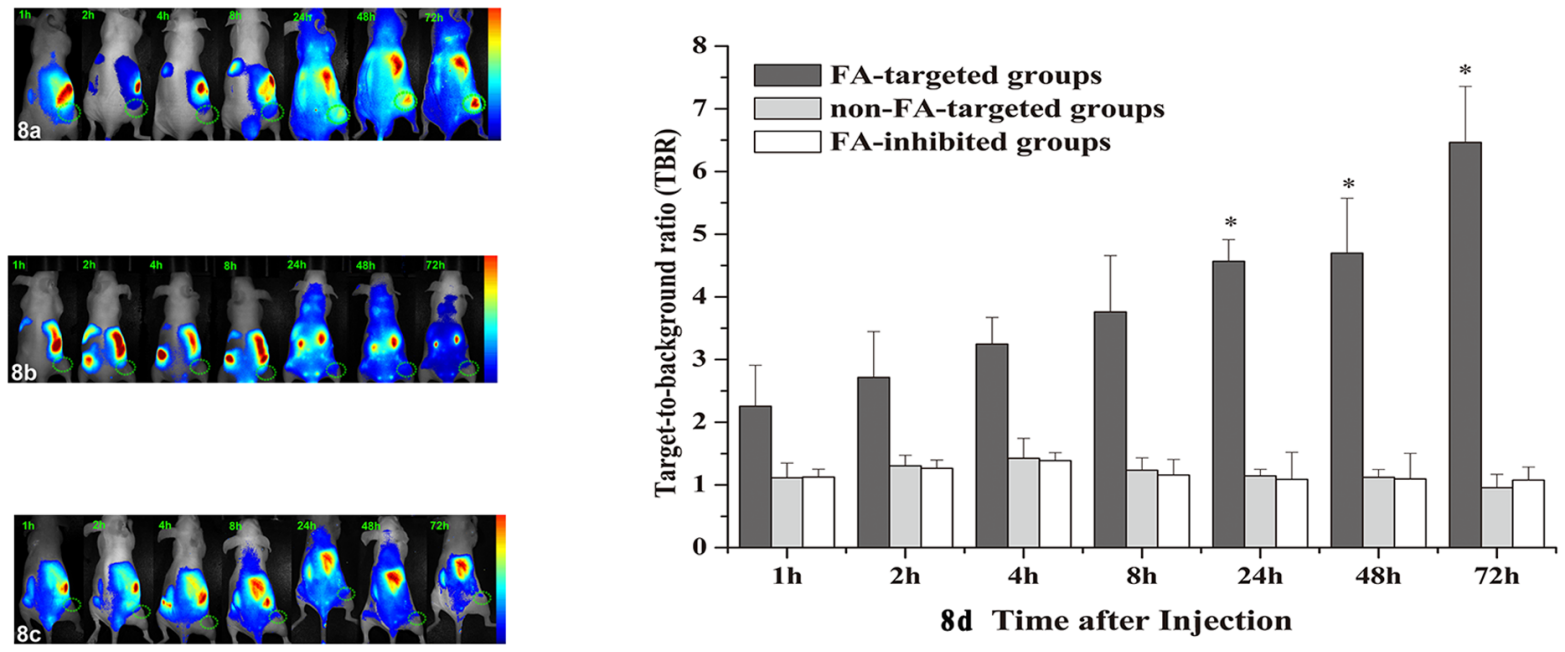
In vivo NIR imaging of three groups at different time intervals after administration of (a) FA-NIR 797-MAN, (b)NIR 797-MAN and (c) FA-NIR-797-MAN+FA. The tumors were circled with dotted line. (The circular indicates the tumor region). (d) Target-to-background ratio (TBR) of FA-targeted group, non-FA-targeted group and FA-inhibited group at different post-injection time. The data are shown as mean ± SD (n = 3), * indicated *P*<0.05.

To investigate the tumour-targeting capabilities of the three groups, nude mice bearing KB tumour xenografts in their right lower limb were used. To determine the *in vivo* biodistribution of the nanospheres, thoracotomy was performed on the mice and fluorescence images were obtained. Figure 8 shows three series of *in vivo* images obtained by the NIR fluorescence imaging system at different times following administration of the FA-targeted (Figure 8a), the non-FA-targeted (Figure 8b), or the FA-inhibited nanospheres (Figure 8c), respectively. After systemic injection through the tail vein, there was no intratumoural accumulation of any of the nanospheres at 2, 4, and 8 h, but the fluorescence intensity within tumours of mice injected with FA-NIR 797-MAN was increased significantly at 24, 48 and 72 h. In contrast, at the same time points, no tumour fluorescence accumulation was observed in mice injected with NIR 797-MAN or FA-NIR-797-MAN+FA. It is worth noting that FA-NIR 797-MAN itself possesses the capability of targeting KB tumour xenografts. Fluorescence intensity of the tumours and muscles was analyzed at different time intervals in order to semi-quantitatively analyse the targeting ability of the nanospheres (Figure 8d). The average target-to-background ratio (TBR) between tumour and adjacent normal muscle were 4.57, 4.70, 6.46 at 24, 48, and 72 h in the FA-targeted groups; 0.94, 1.18, 1.15 1.56 at 24, 48, and 72 h in the non-FA-targeted groups and 0.93, 1.09, 1.07 at 24, 48, and 72 h in the FA-inhibited groups; respectively. These differences were statistically significant (*P*<0.05).

**Figure 9:**
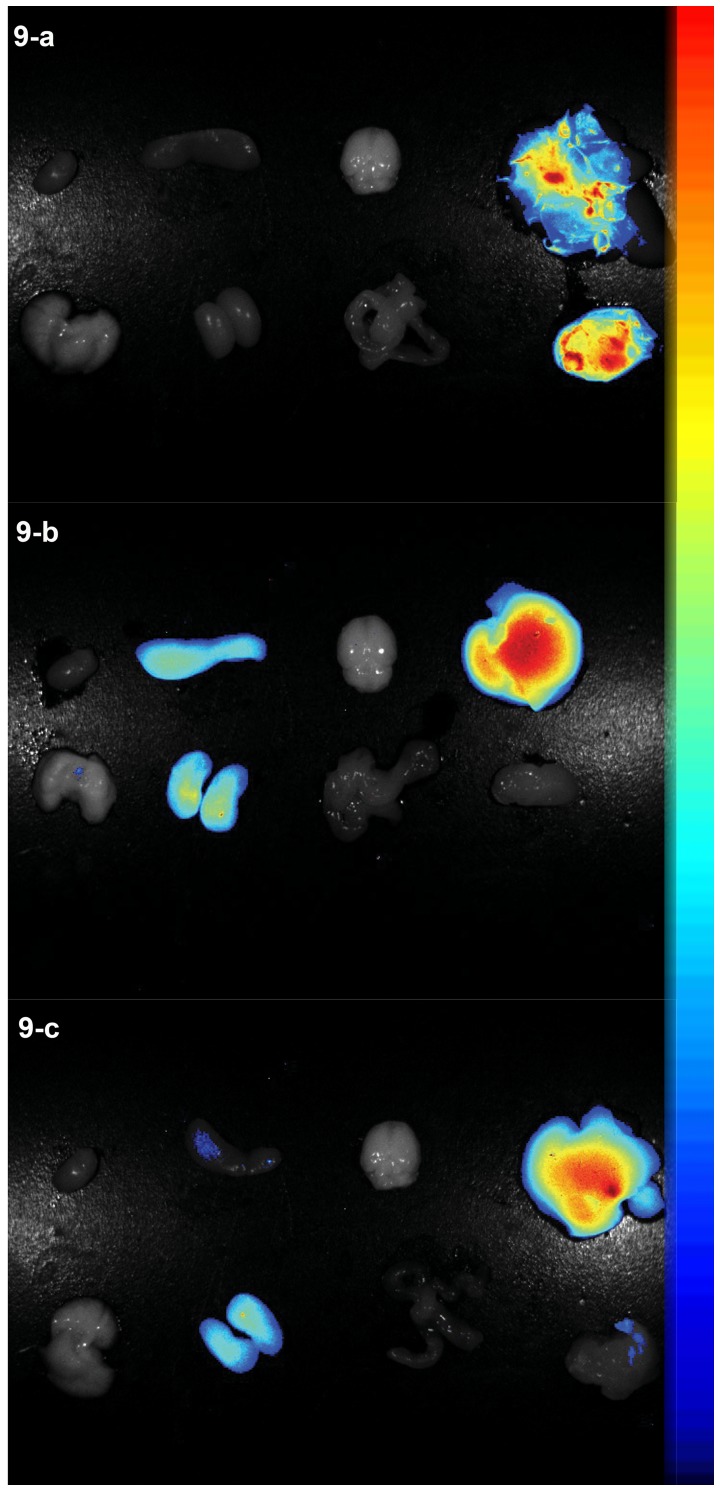
Ex vivo NIR imaging of three groups at 72 h intervals after administration of (a) FA-NIR 797-MAN, (b) NIR 797-MAN and (c) FA-NIR-797-MAN+FA.

To further validate the fluorescence signals in different tissues, the tumour-bearing mice were sacrificed at 72 h post injection, and the tumour, brain, heart, liver, spleen, lung, kidney and intestine were excised, washed with saline (5%), and subjected to fluorescence imaging. As shown in Figure 9 for the *ex vivo* fluorescence imaging of the FA-targeted group (Figure 9a), the fluorescence signal in the tumour was very strong, whereas little signal was visible in the non-FA-targeted (Figure 9b) or FA-inhibited group (Figure 9c). To semi-quantitatively analyse the targeting ability of the probes, the fluorescence intensity in specific regions of interest in the tumours was measured. The peak average fluorescence signal intensities of the tumours were (4.19±1.01)×10^−2^, (1.04±2.26)×10^−4^, and (1.16±1.21)×10^−4^ scaled counts/s/cm^2^ for the FA-targeted, non-FA-targeted, and FA-inhibited groups, respectively; these differences were statistically significant (*P*<0.05). These results were consistent with the findings from the *in vivo* fluorescence images.

NIR 797, an NIR organic dye, was also conjugated to MAN, which facilitated *in vivo* imaging of deep tissues in living subjects by NIR fluorescence imaging. In contrast to the NIR 797-MAN, the FA-NIR 797-MAN nanospheres possess a long circulatory half-life in living subjects and a long retention time in tumours (Figure 8). This suggested the possibility of a sustained anti-tumour drug release to tumours, which minimises the need for administration of several doses of the drug-conjugated probes. The *in vivo* tumour imaging results demonstrated that FA-NIR 797-MAN exhibited relatively high tumour-targeted distribution in FR-positive tumours, while NIR 797-MAN and FA-NIR-797-MAN+FA exhibited relatively low tumour-targeted distribution in FR-positive tumours. Similarly, FA-NIR 797-MAN showed a long retention time in tumour sites (more than 3 days). The *in vivo* study indicated that FA modification improved the tumour-targeting capability of MAN. The *ex vivo* investigation of fluorescence signals in various tissues further revealed tumour-specific accumulation of FA-NIR 797-MAN. Thus, owing to its low toxicity, high NIR fluorescence intensity, and specific tumour-targeting capability, FA-NIR 797-MAN could be utilised as a novel contrast agent for tumour diagnosis, monitoring of the response to therapy, and assessment of prognosis.

### Histology

**Figure 10:**
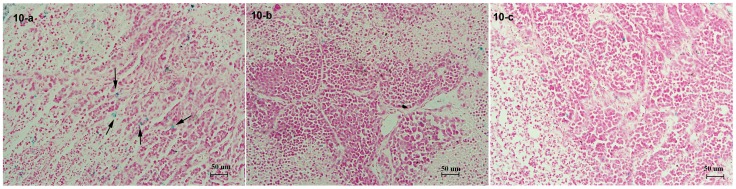
Prussian blue-stained in tumor tissue of nude mice with nasopharyngeal carcinoma after administration of (a) FA-NIR 797-MAN, (b) NIR 797-MAN and (c) FA-NIR-797-MAN+FA at 72 h injection (×200 magnification) (the arrow indicates the blue-stained iron particles).

To further verify the NIR results and confirm the presence of FA-NIR 797-MAN in tumour tissues, tumour slices were resected at 72 h after the injection of FA-NIR 797-MAN, NIR 797-MAN, and FA-NIR-797-MAN+FA, stained successively by Prussian blue for ferric ions and Nuclear Fast Red for the cell nucleus and visualised via optical microscopy. As shown in Figure 10-a, FA-NIR 797-MAN accumulation in tumour tissues was evident from the Prussian blue staining; large aggregates were also visible. By contrast, little blue colour was detected in samples treated with NIR 797-MAN (Figure 10-b) or FA-NIR-797-MAN+FA (Figure 10-c). This result is consistent with the *in vivo* NIR image analyses.

## Materials and Methods

### Materials

All reagents and solvents were purchased commercially and used without further purification. Bovine serum albumin (BSA, fraction V, pH 7.0), folic acid (FA), NIR797 isothiocyanate, N-hydroxysuccinimide (NHS), N-dicyclohexylcarbodiimide (DCC), and 3-(4,5-dimethylthiazol-2-yl)-2,5-diphenyltetrazolium bromide (MTT) were purchased from Sigma-Aldrich. Superparamagnetic Fe_3_O_4_ nanoparticles were prepared via the coprecipitation method as described previously [27], [28]. Isoflurane was purchased from Shandong Keyuan Pharmaceutical Co., Ltd. (China).

### Activation of FA (formation of NHS-folate)

This procedure was carried out as described by Saxena et al. [29] with certain modifications. FA (0.5 g) was first activated with NHS (2.6 g) and DCC (4.8 g) in anhydrous DMSO in the presence of 2.5-ml triethylamine as a catalyst, incubated in a nitrogen atmosphere overnight. The solution was filtered to remove the dicyclohexylurea by-product and then precipitated in cold anhydrous ether. The product was maintained in the dry state following several steps of ether washing, decantation, and vacuum drying.

### Synthesis of magnetic albumin nanospheres (MAN)

The magnetic albumin nanospheres were prepared by a desolvation-crosslinking technique. BSA (250 mg) and superparamagnetic Fe_3_O_4_ nanoparticles (50 mg) were dissolved in 25 mL of purified water. The pH was adjusted to 8.0. Nanospheres were formed by the gradual addition of 100 mL of ethanol with continuous stirring (500 rpm) at room temperature. A pumping device controlled the addition of ethanol at a set rate of 1 mL/min. After the desolvation process, 50 µL of 0.25% glutaraldehyde solution were added to induce particle crosslinking, and the crosslinking process was performed with overnight stirring of the suspension. The suspension was purified by three cycles of centrifugation (22,000 rpm, 30 min) and redispersed in 3.0 mL of purified water.

### Synthesis of folate-conjugated MAN (FA-MAN)

NHS-folate (40 mg) was dissolved in 1.0 mL of anhydrous DMSO and added slowly to the stirring MAN suspension (3 mL, pH adjusted to 10.0). After 2 h of stirring at room temperature, the reaction mixture was passed through a Sephadex G-25 column to separate the FA-MAN from unreacted FA and other by-products. The FA-MAN was eluted in the void fraction. The suspension was purified by three cycles of centrifugation (22,000 rpm, 30 min) and redispersed in 3.0 mL of purified water.

### Synthesis of folate-NIR 797-conjugated MAN (FA-NIR 797-MAN)

NIR 797 (2 mg) was dissolved in 0.5 mL of anhydrous DMSO and added slowly to the stirring FA-MAN suspension (pH adjusted to 8.0). After stirring overnight in the dark at room temperature, the resulting mixture was purified by three cycles of centrifugation (22,000 rpm, 30 min) then subjected to dialysis (MWCO 3500) for 2 days. Finally, the mixture was redispersed in 3.0 mL of purified water. The synthesis and purification of NIR 797-MAN were as described for MAN/FA-MAN.

### Characterisation

UV-Vis spectra were assessed on a Shimadzu UV-3600 spectrophotometer. The iron contents of the FA-NIR 797-MAN were determined by the colourimetric method using *o*-phenanthroline. Transmission electron microscopic (TEM) analysis was carried out on a JEM-2000EX microscope. The hydrodynamic diameter and size distribution of the FA-NIR 797-MAN were determined by dynamic light scattering (DLS) using a Broolhaven BI9000AT system (Brookhaven Instruments Corp.) within 7 days. All measurements were repeated three times at a wavelength of 658.0 nm, and all results were averaged over five runs. Magnetic properties were determined with a vibrating sample magnetometer (VSM, Lakeshore 7407) at room temperature in a magnetic field up to 10 kOe.

### Cell culture

The KB human nasopharyngeal epidermal carcinoma cell line was purchased from the Institute of Biochemistry and Cell Biology, Shanghai Institute of Biological Sciences, Chinese Academy of Sciences. The KB human nasopharyngeal epidermal carcinoma cell line (FR- overexpressing cells) were grown in folate-free RPMI 1640 medium (Invitrogen, IL) and supplemented with 10% foetal bovine serum (FBS), 10 U/mL penicillin, and 10 mg/mL streptomycin. Cultures were maintained at 37°C under humidified conditions with 5% CO_2_. Prior to beginning the experiments, the cells were grown to confluence.

### Cell toxicity assays

KB cells were seeded in 96-well plates at 5×10^3^/well and allowed to adhere overnight. The growth medium was replaced with media containing various concentrations of FA-NIR 797-MAN and NIR 797-MAN. Cells were then incubated for 24 h and washed three times with 1 mL of PBS. Subsequently, cells were incubated in growth media containing 1 mg/mL MTT agent for an additional 4 h at 37°C, and 500 µL of DMSO were added to each well to solubilise the formazan crystals. The samples were tested in triplicate. The optical density (OD) of the solution at 490 nm was measured with a microplate reader, and cell viability was calculated by the following equation: viable cells (%)  =  (OD treated/OD control) ×100%, where OD treated was the measurement from the samples incubated with FA-NIR 797-MAN and NIR 797-MAN, and OD control was the measure of the incubation medium.

### Nanosphere uptake by KB cells

KB cells were cultured in a 24-well plate at 10^5^/well. To compare nonspecific uptake, FA-NIR 797-MAN (FA-targeted group), NIR 797-MAN (non-FA-targeted group), and FA-NIR 797-MAN with the addition of free FA (FA-NIR 797-MAN+FA) (FA-inhibited group) were added to a final concentration of 25 µg/mL Fe per well. After 24 h, each well was washed three times with PBS, treated with 0.5 mL of 4% paraformaldehyde for 30 min to fix the cells, and then washed with PBS. For staining of intracellular iron, 2 mL of Prussian blue solution, comprising equal volumes of 2% aqueous hydrochloric acid and 2% potassium ferrocyanide (II) trihydrate, were added to the fixed cells for 30 min. After washing with PBS, cells were mounted under a microscope for observation.

### Animal models

BALB/c nude mice were purchased from the Academy of Military Medical Science (China).All experiments involving animals were performed in compliance with the guidelines of the Animal Care Committee of the Southeast University Nanjing, China. All animals received humane care in compliance with the ‘Principles of Laboratory Animal Care’ formulated by the National Society for Medical Research and the ‘Guide for the Care and Use of Laboratory Animals’ prepared by the Institute of Laboratory Animal Resources and published by the National Institutes of Health (NIH Publication No. 86-23, revised 1996).

For tumour implantation and optical imaging, animals were anesthetised with 1.0% isoflurane in a head holder through a nosecone, and respiration rates were monitored. KB cells (2×10^6^ per mouse) were inoculated subcutaneously into the right lower limb of nude mice (6–8 weeks, 22–26 g). All mice received a folate-deficient diet for 3–4 weeks prior to optical imaging in order to reduce the serum folate concentration to human physiological levels. The tumours were imaged upon reaching a size of ∼0.5 cm.

### *In vivo* optical imaging

The FA-targeted, non-FA-targeted, and FA-inhibited groups were each intravenously injected into the tail vein of mice. Eight mice were examined per group. After i.v. administration, the time-dependent biodistribution in tumour-bearing mice was imaged using the Maestro *in vivo* optical imaging system (excitation: 795 nm, emission: 814 nm, exposure time: 500 ms; Caliper Life Sciences, MA, USA). The fluorescence images were then analysed based on their spectral patterns using the Maestro 2.10.0 software (Caliper Life Sciences, MA, USA). Scans were performed at 1, 2, 4, 8, 24, 48 and 72 h post i.v. administration, after which tumour-bearing mice were sacrificed. Tumours, heart, brain, liver, spleen, kidneys, intestines, and lungs were harvested and imaged to estimate the tissue distribution of the nanospheres. To compare the targeting ability of the FA-targeted, non-FA-targeted, and FA-inhibited groups, target-to-background ratio (TBR) at different time points were calculated by using regions of interest (ROI) functions of the Maestro 2.10.0 software. Circular ROIs were selected manually by drawing regions on the *in vivo* optical images. The TBR was calculated using the following formula: TBR  =  SI_T_/SI_M_, where SI_T_ is the average signal of the tumor and SI_M_ is the average signal of the contralateral thigh muscle. This procedure was carried out as described by Alencar et al. [30].

### Histology

At 72 h following the injection of the FA-targeted, non-FA-targeted, and FA-inhibited groups, the tumour tissues from the mice were dissected and fixed in 10% neutral-buffered formalin. The tissues were processed routinely into paraffin, sectioned at a thickness of 4 µm, stained successively by Prussian blue for ferric ions and Nuclear Fast Red for the cell nucleus, and then examined by optical microscopy.

### Statistical analysis

Data are presented as means ± standard deviation. All multiple comparisons were performed by one-way ANOVAs followed by Tukey's post hoc tests. All statistical tests were performed using SPSS for Windows (Version 13.0; SPSS), and a value of *P*<0.05 was considered to indicate statistical significance.

## Conclusions

In conclusion, a practical and effective approach for synthesising FA-NIR-797-MAN was developed. In this approach, MAN were covalently conjugated with FA ligands to enhance the targeting capability of the particles to FR over-expressing tumours. NHS-folate was conjugated onto MAN with the amide linker to yield FA-MAN, which was then incorporated with NIR 797 via an amide linker to yield FA-NIR 797-MAN. The synthesised FA-NIR 797-MAN were characterised by UV-Vis, TEM, DLS and VSM, demonstrating the covalent linkage of FA and NIR 797, the high solubility and stability of the FA-NIR 797-MAN in an aqueous medium, and the stable magnetic properties of the FA-NIR 797-MAN, respectively. The FA-NIR 797-MAN exhibited no toxicity to KB cells *in vitro*. Compared with the NIR 797-MAN and FA-NIR-797-MAN+FA, the FA-NIR 797-MAN exhibited enhanced uptake by FR-positive KB cells. *In vivo*, the FA-NIR 797-MAN displayed higher tumour targeting capability to FR-positive tumours than did the NIR 797-MAN or the FA-NIR-797-MAN+FA. Our data suggest that FA-modified NIR 797-MAN has considerable potential for early tumour diagnosis and targeted therapy. We have shown that near-infrared fluorochromes can be modified by small molecules other than peptides and can be used for targeting of receptor systems. These probes and their modifications will be useful for more extensive biological and medical applications.
